# First evidence of recombinant Myxomavirus (ha-MYXV) in European hare (*Lepus europaeus*) in the Czech Republic and Slovakia

**DOI:** 10.3389/fvets.2026.1754249

**Published:** 2026-01-23

**Authors:** Kamil Sedlák, Kateřina Mikulášková, Alexander Nagy, Jan Cukor, Pavla Príhodová, Lenka Černíková, František Kostka, Petr Václavek

**Affiliations:** 1 State Veterinary Institute Praha, Praha, Czechia; 2 State Veterinary Institute Jihlava, Jihlava, Czechia; 3 Forestry and Game Management Research Institute, Jíloviště, Czechia; 4 Faculty of Forestry and Wood Sciences, Czech University of Life Sciences Prague, Prague, Czechia

**Keywords:** Central Europe, lagomorphs, mortality, myxomatosis, recombinant virus

## Abstract

Recombinant myxoma virus (ha-MYXV) is an important causative agent of a fatal disease affecting hares (*Lepus* spp.). It was first identified in the Iberian Peninsula in 2018 and it subsequently spread to Western Europe during 2023–2024. Here, we report the emergence of this severe disease in the Czech Republic and Slovakia. The ha-MYXV was first detected in a European hare (*Lepus europaeus*) found dead in the Czech Republic on August 25, 2025. By the end of September, a total of 21 cases had been confirmed, 20 in European hares and one in a European rabbit (*Oryctolagus cuniculus*). In Slovakia, the virus was first detected in a hare found dead on October 9, 2025, followed by three additional cases. Our findings indicate the ongoing expansion of ha-MYXV, supported by evidence of cross-species transmission and an increasing geographic distribution to other Central European countries, with a projected fatal impact on European hare (*Lepus europaeus*) populations, as previously observed on the Iberian Peninsula.

## 1 Introduction

Myxomatosis in hares is a newly emerging disease that has given rise to concerns regarding its impact on wild lagomorph populations. Classical myxoma virus (MYXV) causes widespread severe disease in domestic and European rabbits (*Oryctolagus cuniculus*) in Europe, and except for a few sporadic cases (1) it does not pose a serious threat to European hare (*Lepus europaeus*) populations. However, in 2018, fatal myxomatosis in Iberian hares (*Lepus granatensis*) caused by a hare-adapted natural recombinant MYXV (ha-MYXV) was discovered in Spain (2) and Portugal (3). This novel virus (designated as Toledo strain) originated through recombination between the classical MYXV and an unidentified poxvirus (4).

The ha-MYXV infection is mainly characterized by bilateral blepharoconjunctivitis, sometimes accompanied by purulent discharge, as well as epistaxis. Typical findings included oedema of the nasal, oral, genital, and anal orifices. Severe and generalized congestion of internal organs, particularly the lungs, together with pulmonary oedema and pronounced haemothorax, were also frequently observed (5).

The spread of ha-MYXV across the Iberian Peninsula led to high mortality in hare populations (6). In August 2024, ha-MYXV was reported near the German-Dutch border, specifically from North Rhine-Westphalia (Germany) and Overijssel and Gelderland (Netherlands). However, time-aware phylogenetic analysis suggested that the virus had already been circulating in this region between 2020 and 2023, with the potential for further expansion (7). Consequently, in 2025, the ha-MYXV was detected in Austria (8). Here, we present the first evidence of ha-MYXV in the Czech Republic and Slovakia.

## 2 Materials and methods

### 2.1 Samples

All of the animals involved in this study were found dead with lesions suggestive of myxomatosis in the South Moravian Region, Czech Republic, and were sent for postmortem examination from August 25, to September 25, 2025. The carcasses underwent gross examination, and suspicious skin lesions were examined histopathologically in three hares. Samples from the eyelids, nostrils, lips, and organs of all 16 hares and one European rabbit were collected for molecular detection and discrimination of ha-MYXV. A total of nine samples of suspensions from the skin of the eyelids and lungs of hares were examined by isolation on cell cultures. Moreover, between October 15 and 21, four dead hares from the Trnava region in southwestern Slovakia were submitted for MYXV testing. The shortest distance between the tested carcasses in the Czech Republic and Slovakia was approximately 85 km.

### 2.2 Detection of the Czech ha-MYXV strain

Total nucleic acid was extracted from 200 μl of organ pool supernatant by MagNA Pure 24 (Total NA Isolation Kit, Roche) and eluted into 50 μl. The ha-MYXV was detected and typed by a multiplex qPCR assay (QuantiTect Probe PCR kit, Qiagen) for detection and differentiation between the classical and ha-MYXV variants (6).

### 2.3 Virus isolation

Virus isolation was performed from homogenized samples of parenchymatous organs and skin lesions, following the procedures of Salem et al. (9) and WOAH Terrestrial Manual (10) with minor modifications. Two cell lines, RK-13 (rabbit kidney) and VERO (African green monkey kidney), were used in parallel. Tissue homogenates (0.1 g in 1 ml of MEM supplemented with 2% FCS, antibiotics) were prepared using a Precellys Evolution homogenizer, centrifuged, and filtered through a 0.45 μm pore-size membrane filter. Cell monolayers were inoculated with 300 μl of the supernatant and incubated at 37 °C in a 5% CO_2_ atmosphere. After a 2-h adsorption period, the inoculum was replaced with a fresh medium (MEM, 2% FCS, antibiotics), and the cultures were monitored daily for cytopathic effect (CPE) for up to 7 days. Samples showing no CPE were frozen and subjected to a second passage. The first CPE was detected on day 6 post-inoculation in one skin lesion sample on VERO cells, with a distinct CPE observed within 5 days after reinoculation. The virus was harvested by freezing the infected cell culture and subsequently confirmed by sequencing.

### 2.4 Whole genome sequencing

For the WGS, we used a modified SISPA (Sequence Independent Single Primer Amplification) approach (11). First, the virus culture was spined (14,500 rpm/5 min) and 200 μl of supernatant was treated with Saponin (2% final concentration) and incubated at room temperature for 5 min. Next, the reaction volume was adjusted by ddH_2_O to 450 μl and treated with TURBO Dnase (Thermo Fisher) in two consecutive steps, each with 3 μl of enzyme, incubated for 30 min at 37 °C. Finally, nucleic acid was extracted from 300 μl of treated virus culture (Maxwell RSC, Pathogen Total Nucleic Acid Kit, Promega) and eluted in 100 μl. The SISPA amplification was prepared according to an in-house protocol (available on request).

The sequencing libraries were purified by SPRIselect magnetic beads (Beckman-Coulter) and quantified by QIAxpert (Qiagen). End preparation, native barcoding, and sequencing adapter ligation were performed by Native Barcoding Kit [SQK-LSK-114.96, Oxford Nanopore Technologies (ONT)], according to the manufacturer's instructions. Sequencing was carried out on the ONT MinION Mk1D using R10.4.1 flow cells and operated via MinKNOW software (v2.3.04.3, ONT). Basecalling was conducted with Dorado (v0.5.0, ONT) using the super accurate basecalling model (dna_r10.4.1_e8.2_400bps_sup@v4.3.0 model). The WGS run was monitored in real time by the Read Assignment, Mapping, and Phylogenetic Analysis in Real Time (RAMPART) module of the ARTIC bioinformatic pipeline (Artic Network) set to the ha-MYXV genome as a reference. Demultiplexing and adapter and barcode trimming were performed by Porechop and Dorado. Consensus sequence was generated using ViralConsensus (12) and manually curated against the corresponding SAM files to obtain a final consensus genome. The genomic information of the ha-MYXV strain: Czech_Republic/BV/VI9478_05-Sep-2025 was submitted to the GenBank under the accession code PX512303.

### 2.5 Phylogenetic analysis

MYXV genomes with the highest Basic Local Alignment Search Tool (BLAST) hits were downloaded and aligned with Multiple Alignment using Fast Fourier Transform (MAFFT) (13). Alignment trimming and format conversion (Phylip full names and padded) were performed by AliView (14). Maximum likelihood (ML) tree was calculated using IQ-TREE multicore version 2.2.0-beta for Linux 64-bit (15), with 1,000 replicates. The best-fit model HKY+F was selected based on the Bayesian Information Criterion.

## 3 Results

**Figure 1:**
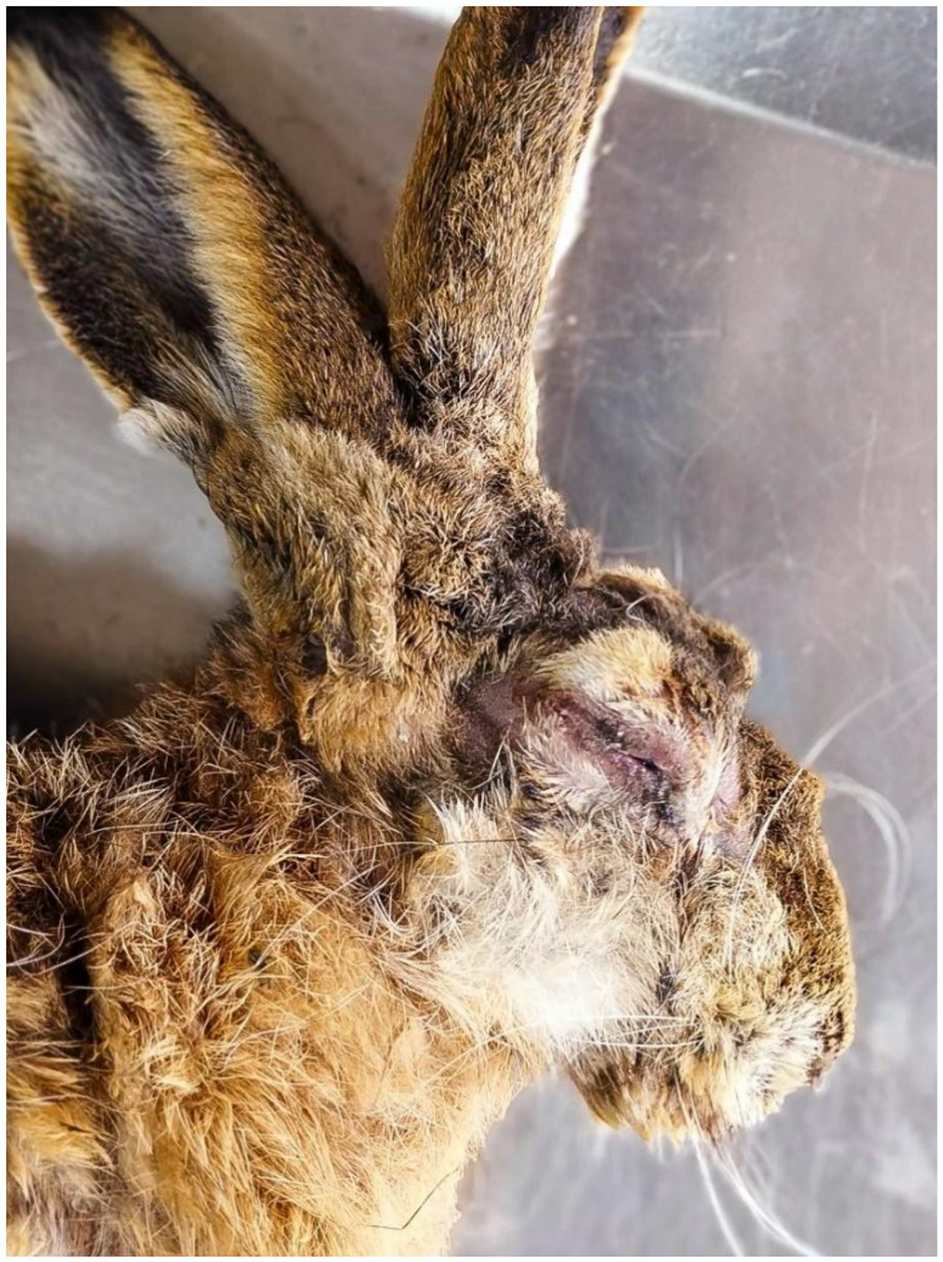
Macroscopic lesions. Head of an infected hare showing mucopurulent discharge from the eyes and nose, purulent ocular discharge, and swelling of the eyelids, lips, and nostrils.

**Figure 2:**
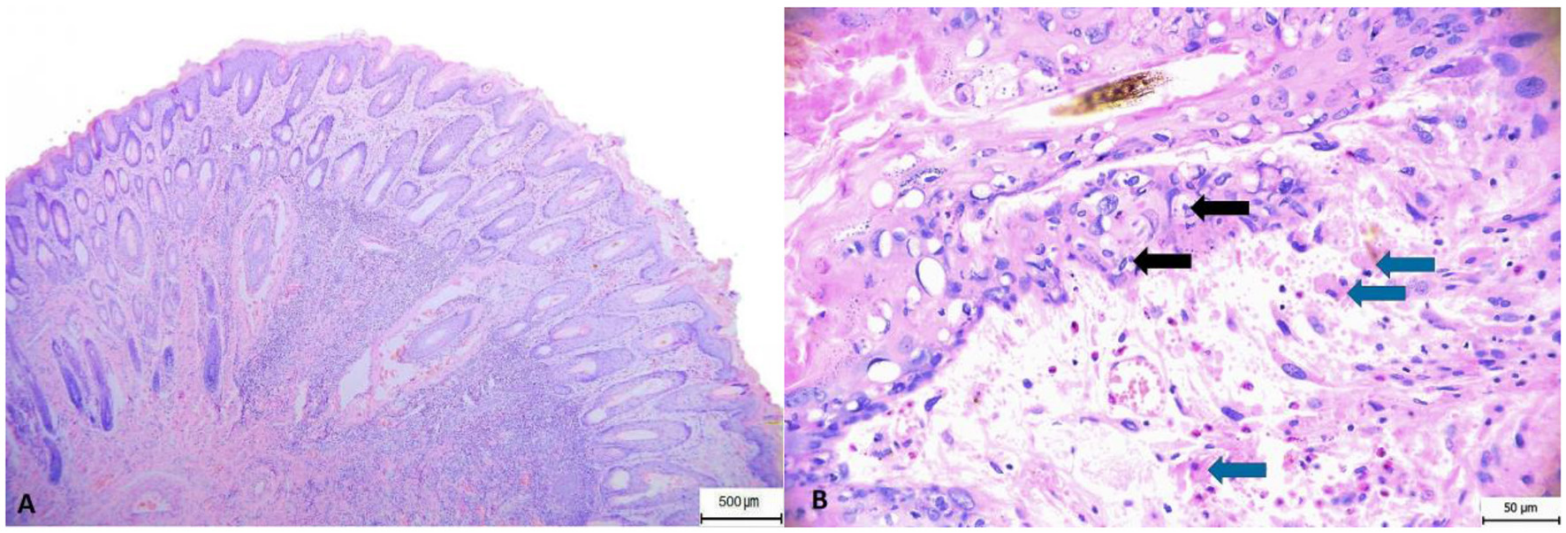
Histopathology. Eyelid skin showing: **(A)** hyperkeratosis, pyogranulomatous infiltration, perifollicular edema and hemorrhage. **(B)** Pyogranulomatous infiltration with admixture of eosinophilic granulocytes; hydropic degeneration. Black arrows = cytoplasmic inclusion bodies; blue arrows = myxomatous cells. Hematoxylin and eosin stain.

We performed pathological examinations on 16 European hares and one European rabbit found dead in the Czech Republic. The hares were adults animals of both sexes. Two (12.5%) hares were markedly wasted, four were moderately emaciated (25.0%), the others were in good to very good nutritional condition (weight 3–5 kg), and the rabbit was in very good nutritional condition (1.4 kg, male). In hares, macroscopic lesions (Figure 1) included cyanosis of visible mucous membranes (5, 31.3%), mucopurulent discharge from the eyes or nose (7, 43.5%), or serohemorrhagic and purulent discharge from the eyes (2, 12.5%), swelling of the eyelids, lips, and nostrils (8, 50.0%), subcutaneous tissue locally swollen and thickened (7, 43.8%), enlarged and congested superficial lymph nodes (4, 25.0%), hyperemia of the upper respiratory tract (8, 50.0%), and hyperemia and edema of the lungs (13, 81.3%). The rabbit had conjunctival hyperemia and swelling of the eyelids, enlargement and congestion of the superficial lymph nodes, and hyperemic edema of the lungs. The histopathological examination of lesions on the eyelids and nostrils revealed epidermal hyperplasia with marked acanthosis and orthokeratotic hyperkeratosis. Furthermore, focal spongiosis and high numbers of cells with glassy-looking cytoplasm and pyknotic nuclei were observed, as well as vacuolar to ballooning degeneration of keratinocytes and isolated intracytoplasmic inclusions. Basophilic myxoid substance and intercellular edema are evident subepithelially, while in the deeper layers of the dermis, especially periadnexally, there are deposits of mixed inflammatory infiltrate with abundant eosinophilic granulocytes. Myxoma cells were found in lower numbers, while macrophages were more abundant (Figure 2).

**Figure 3:**
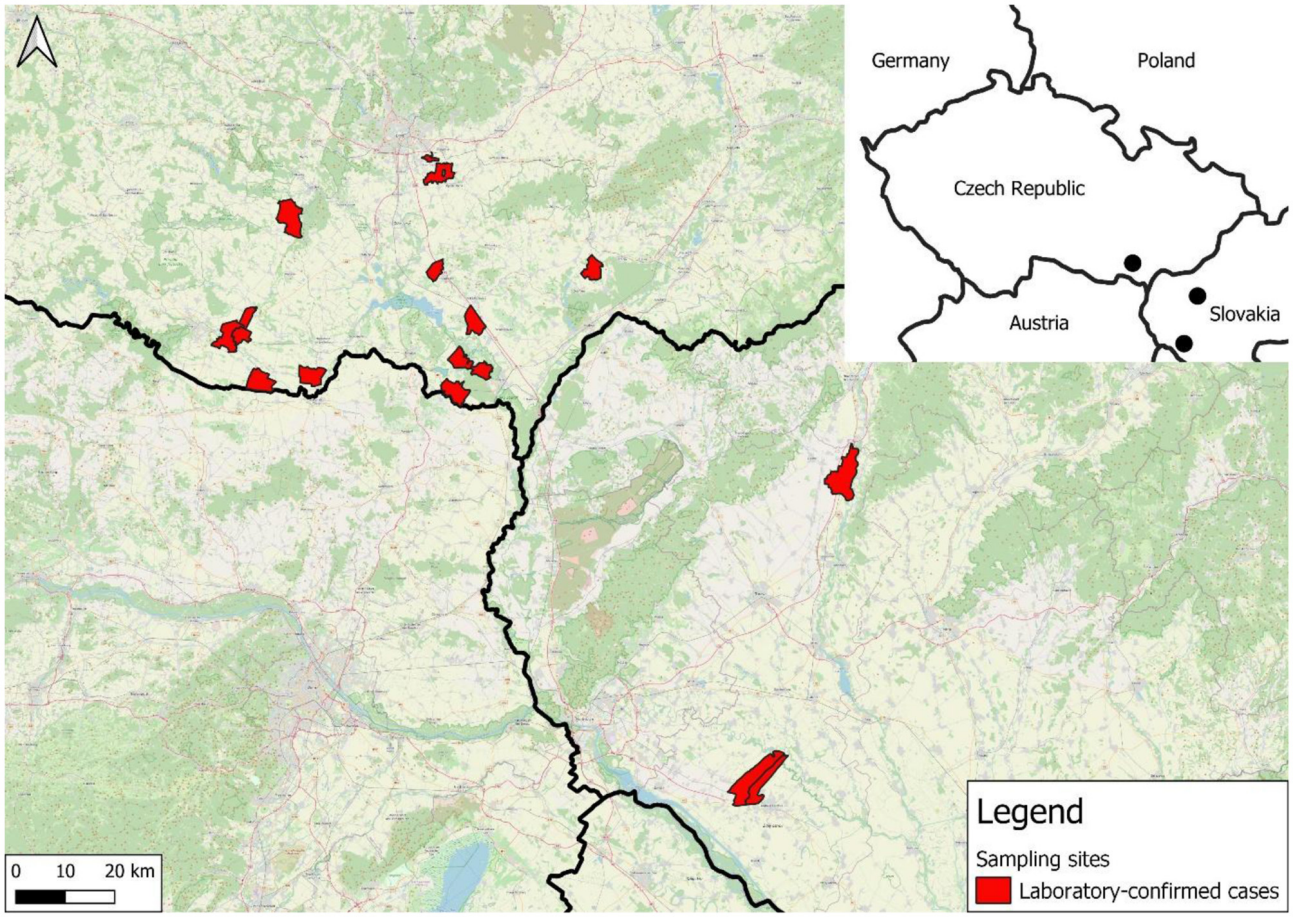
Distribution of confirmed cases of recombinant ha-MYXV according to particular hunting districts.

MYXV was detected in all 16 hares from the Czech Republic and four from Slovakia using qPCR, and recombinant ha-MYXV was confirmed in all of them using a subsequent discriminatory method. One European rabbit was qPCR-positive for MYXV and a follow-up discriminatory assay identified the strain also as ha-MYXV. The distribution of confirmed cases of recombinant ha-MYXV by hunting grounds in which the carcasses were discovered is shown in the accompanying map (Figure 3).

**Figure 4:**
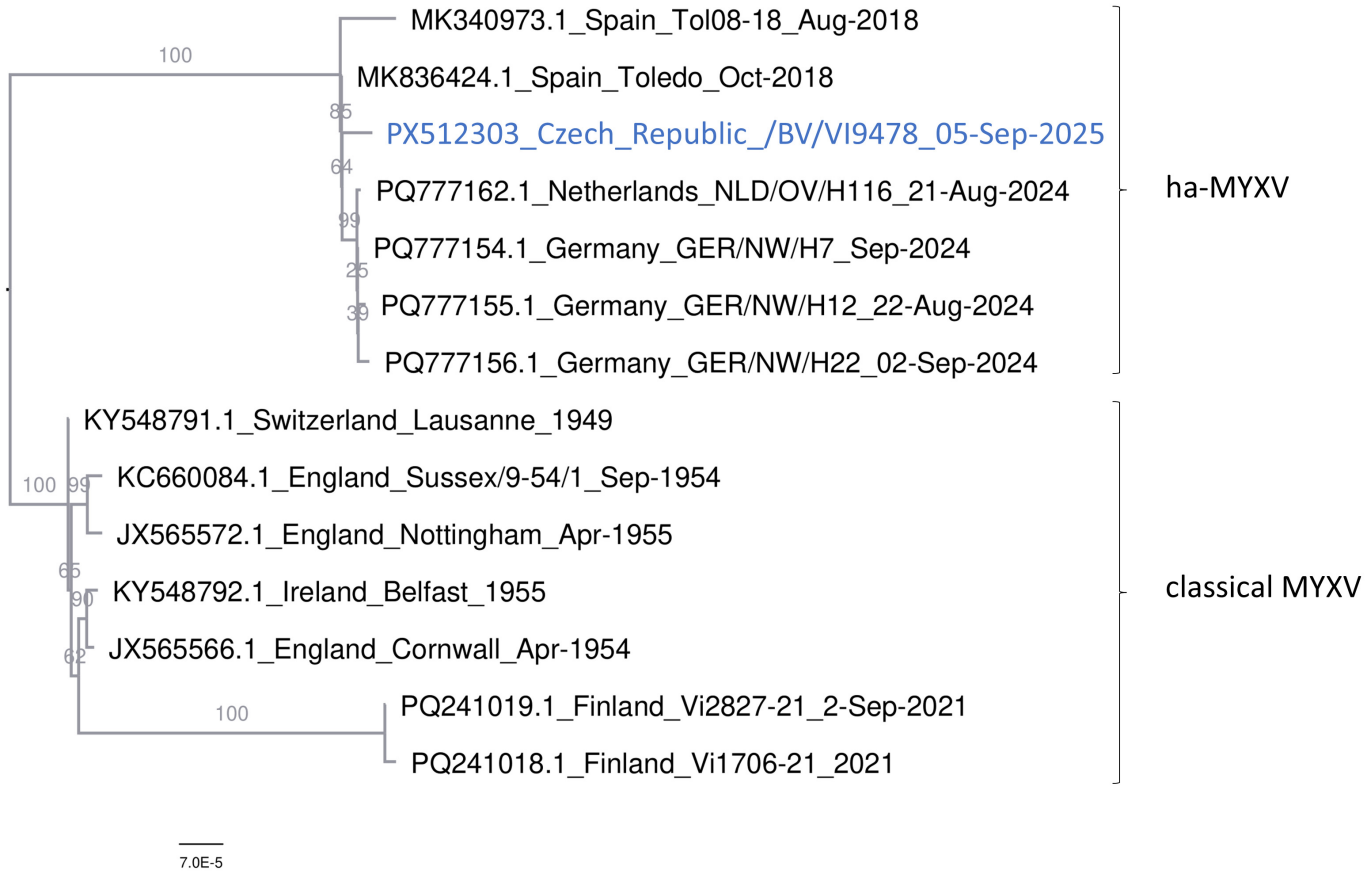
Phylogenetic analysis. An ML tree calculated from the representative European MYXV and ha-MYXV whole genomes. The tree is midpoint-rooted. Bootstrap values, calculated from 1,000 replicates, are shown as percentages at each branch node.

A representative ha-MYXV genome obtained from a cell culture was sequenced by WGS. BLAST search (16) suggested that the Czech ha-MYXV strain showed 99.99% sequence similarity to the Spanish, German, and Dutch ha-MYXV strains detected during 2018–2024. Similarly, a maximum likelihood tree (Figure 4) clearly placed the Czech ha-MYXV strain into the group of these hare-adapted recombinant variants. Sequence comparison suggested that the Czech virus differed from the Spanish Toledo/2018 strain (MK836424) by eight point mutations and one three-nucleotide deletion (Table 1).

**Table 1:** Comparative genomic analysis of Czech and Toledo ha-MYXV isolates.

**Position/difference**	**Gene**	**Change**
13514–13516 ATC codon deletion	Host range protein	Deleted D167 in a poly D stretch
11751 G/A	Putative E3 Ub ligase	Mutation R161C
22941 C/T	Ser/Thr protein kinase	Synonymous [E] GAG/GAA
39888 G/A	Thiol-oxidoreductase	Synonymous [A] GCG/GCA
43087 C/G	Core protein m038L	Mutation E32D
52348 G/A	Core protein m049R	Synonymous [T] ACG/ACA
81767 G/A	Nucleoside triphosphatase	Synonymous [A] GCG/GCA
148224 G/A	E3 Ub ligase	Synonymous [A] GCG/GCA
150412 C/T	Serine protease inhibitor	Mutation P301S

## 4 Discussion

In the present study, we report the occurrence of ha-MYXV in hare populations in the Czech Republic and Slovakia. Our results confirm the ongoing spread of this infection to novel geographic regions into the Central Europe.

It is difficult to infer the origin of the Czech ha-MYXV in greater detail due to the limited genomic information available from many affected regions. We hypothesize that the infection to the Czech Republic was introduced relatively recently from Austria (8) (or possibly Slovakia) suggesting that Central Europe represents a newly affected area. This assumption is supported by the geographical proximity of outbreak localities to the Austrian border, as well as by the phylogenetic analysis, in which the Czech isolate clustered separately from the German strains and showed closest relationship to the Toledo strain. Given these distinctions, it is reasonable to consider a south-to-north transmission pathway, independent on the German outbreaks. Nevertheless, this conclusion should be validated by phylogenetic analysis of Austrian ha-MYXV genomic sequences.

The detection of ha-MYXV-positive hare carcasses in the Czech Republic and Slovakia during late summer and autumn 2025 suggests that local transmission may have occurred via arthropod vectors, similar to the spread mechanisms of classical MYXV.

Our findings confirm previous results that ha-MYXV is highly lethal in hares (17). However, the impact of this infection to the Central European hare population remains to be determined. In any case, the ha-MYXV virus will represent a new challenge for the entire species in the European context. In assessing the impact of myxomatosis on hare populations, overall mortality is a key indicator. In the Iberian hare, mortality was estimated at approximately 57%, based on data from southern Spain, where around 530 dead individuals were found in outbreak areas in 2018 (2). Subsequent monitoring between 2019 and 2021 across 178 hunting districts revealed a rapid population decline from 12.7 hares/100 ha in 2019–2020 to 4.7 hares/100 ha in the following season. This farmland species has already been experiencing a long-term population decline primarily caused by changes in agroecosystems (18), and according to current knowledge, the recombinant ha-MYXV virus is expected to significantly influence its populations in the affected areas over the coming decades. Nevertheless, it is necessary to emphasize the absence of a standardized monitoring system for ha-MYXV across Europe. The currently confirmed occurrences do not necessarily indicate that the virus is absent from regions where it has not yet been detected, but rather may reflect a lack of available data. Therefore, further spread or the confirmation of new occurrences can be expected (7), and it is highly advisable to pay close attention to this disease even in areas where it has not yet been confirmed.

## Data Availability

The original contributions presented in the study are publicly available. This data can be found here: The genomic information of the ha-MYXV strain: Czech_Republic/BV/VI9478_05-Sep-2025 was submitted to the GenBank under the accession code PX512303.
